# The Burden of Hypertension and Diabetes Mellitus and Their Predictors in an Urban Slum of Chhattisgarh, India: A Retrospective Record-Based Study

**DOI:** 10.7759/cureus.80953

**Published:** 2025-03-21

**Authors:** Ashish W Khobragade, Manisha M Ruikar, Gaurav Singh, Anupriya Jha

**Affiliations:** 1 Department of Community and Family Medicine, All India Institute of Medical Sciences, Raipur, IND

**Keywords:** burden, diabetes, hypertension, non-communicable diseases, urban slum

## Abstract

Background

Non-communicable diseases pose a significant health burden in India. Hypertension and diabetes are known to affect underprivileged communities in terms of healthcare costs disproportionately. This study aimed to determine the burden of these two diseases and their predictors in an urban slum of Raipur city.

Methods

We conducted a retrospective record-based study in an urban slum of Raipur. A population-based screening register is maintained in the Urban Health Training Centre, All India Institute of Medical Sciences (AIIMS), Raipur. Data about adults who underwent screening from January 2023 to December 2023 were extracted from this register. Sociodemographic details, lifestyle factors, family history of NCDs, and medication history were collected and analyzed. The prevalence of diabetes mellitus and hypertension was estimated, and logistic regression was performed to identify the important risk factors for diabetes and hypertension.

Results

Out of 1,005 study participants, the median age was 48, with an IQR of 38-60 years. The prevalence of hypertension and diabetes mellitus was 33% and 16%, respectively. In logistic regression, age (adjusted odds ratio (aOR): 1.06, 95% CI: 1.05-1.08), alcohol consumption (aOR: 2.98, 95% CI: 1.13-7.85), and diabetes (aOR: 2.55, 95% CI: 1.75-3.70) were significant risk factors for hypertension. For diabetes mellitus, age (aOR: 1.03, 95% CI: 1.01-1.04) and hypertension (aOR: 2.57, 95% CI: 1.77-3.75) were significant risk factors.

Conclusions

We found a high prevalence of hypertension and diabetes mellitus in the urban slum, with increased risk with increasing age, alcoholics, and those with comorbid conditions. Early and targeted intervention, focusing on modifiable lifestyle factors and early screening of comorbid conditions, is crucial for managing and reducing the burden of non-communicable diseases in vulnerable communities.

## Introduction

Hypertension and diabetes mellitus are the two leading non-communicable diseases (NCDs) that contribute substantially to health burdens and out-of-pocket expenditures [1]. They substantially increase the risk of cardiovascular diseases, cerebrovascular accidents, retinopathy, neuropathy, and nephropathy. Both chronic conditions are part of a constellation of metabolic derangements called metabolic syndrome, which increases the risk of several complications [2]. Nearly three-fourths of deaths globally are due to NCDs [3]. A similar burden is seen in India, with 60% of all mortalities due to noncommunicable diseases. Due to the epidemiological transition, the burden of NCDs has increased in India [4,5].

Rapid urbanization in India has led to a significant increase in the population of people living in urban slums. India’s urban population is expected to reach 40% by 2030 [6]. This has also led to a mix of changes in socio-cultural settings characterized by poor living conditions, overcrowding, faulty feeding practices, lack of exercise, drug abuse, and poor socioeconomic status. Urban slum areas often lack access to healthcare facilities, both in terms of availability and accessibility. All these factors increase the risk of noncommunicable diseases [6,7].

National Family Health Surveys (NFHS) have highlighted the increasing burden of hypertension and diabetes mellitus among different demographic groups [8]. However, the data on these in an urban slum setting are limited. Therefore, this retrospective, record-based study aimed to assess the burden of hypertension and diabetes mellitus, as well as their associated risk factors, in an urban slum. The findings from this study may provide valuable insight to address hypertension and diabetes mellitus in urban slums and similar low-resource settings.

## Materials and methods

A retrospective record-based study was conducted to assess the burden of hypertension, diabetes mellitus, and their associated risk factors in an urban slum of Raipur.

Study setting

The study was done in an urban slum of Raipur, Chhattisgarh, India. Population-based screening is conducted regularly in the catchment area of the Urban Health Training Centre (UHTC) at All India Institute of Medical Sciences (AIIMS), Raipur. The total population of this area is approximately 50,000, consisting of two wards. The study utilized medical records from population-based screening registers maintained in UHTC. The data captured in the register are basic sociodemographic details, tobacco and alcohol use, other comorbidities, medication history, and family history of hypertension and diabetes mellitus.

Study population

The study included adults aged 30 years and older who had been screened for diabetes mellitus and hypertension in the community between January 1, 2023, and December 31, 2023. Individuals with incomplete records were excluded.

Survey procedure

Houses were selected randomly from two wards. Those who were not willing to participate in the survey were excluded. Age, sex, history of smoking and alcohol consumption, and history of hypertension and diabetes in the family details were recorded by face-to-face interview. Both systolic and diastolic blood pressure were measured in a sitting and relaxed position for five minutes, with two readings obtained one to two minutes apart, and the average of these two readings was recorded. Random blood sugar levels were measured using a portable glucometer via the finger-prick method. Those who were found to have raised blood pressure and sugar levels were referred to UHTC for further management.

Data collection

We extracted information related to age, gender, occupation, alcohol and tobacco use status, family history of hypertension and diabetes mellitus, systolic and diastolic blood pressure and random blood sugar, and other comorbid conditions. The data were anonymized to maintain patients' confidentiality.

Operational definitions

Hypertension: If the measured systolic blood pressure is ≥140 mmHg and/or diastolic blood pressure is ≥90 mmHg or is already on antihypertensive treatment.

Diabetes mellitus: If the random blood sugar level is ≥200 mg/dL with the presence of symptoms [9] or the person is already on antidiabetic medication.

Risk factors: If a person had consumed alcohol or tobacco in the past month, they were classified as an alcoholic or smoker, respectively.

Statistical analysis

Epicollect5 (Centre for Genomic Pathogen Surveillance Team, Oxford, UK) was used for data extraction [10]. Data were analyzed using SPSS version 20 (IBM SPSS Statistics for Windows, IBM Corp., Armonk, NY). Median, frequencies, and percentages were used to summarize the demographic characteristics and risk factors. The burden of hypertension and diabetes mellitus was estimated with a 95% confidence interval. The Mann-Whitney U test was used to explore the association between demographic factors and hypertension and diabetes mellitus. To adjust for potential confounders, multivariable logistic regression was used to find significant predictors of hypertension and diabetes mellitus.

Ethical considerations

The institutional ethics committee approved this study. It is a retrospective, record-based study that uses de-identified individual data; therefore, a waiver of informed consent was granted. The data were handled confidentially to ensure privacy.

## Results

One thousand five people over 30 years of age were screened in one year. Their median age was 48 years (interquartile range (IQR): 38-60 years). Two-thirds of the study population were females, and 60.5% were unemployed (Table 1).

**Table 1:** Sociodemographic and clinical details of the people screened (n = 1,005)

Variables		Frequency (%)
Age group (years)	30-44	397 (39.5)
	45-64	465 (46.3)
	≥65	143 (14.2)
Sex	Female	669 (66.6)
	Male	336 (33.4)
Occupational status	Employed	397 (39.5)
	Unemployed	608 (60.5)
Family history of hypertension/ diabetes	Yes	38 (3.8)
	No	967 (96.2)
Tobacco consumption (any form)	Yes	267 (26.6)
	No	738 (73.4)
Alcohol consumption	Yes	22 (2.2)
	No	983 (97.8)
Blood pressure (≥140/90 mmHg)	Yes	189 (18.8)
	No	816 (81.2)
Random blood sugar (≥200 mg/dL)	Yes	97 (9.7)
	No	908 (90.3)
On anti-hypertensive medications	Yes	221 (22.0)
	No	784 (78.0)
On anti-diabetic medication	Yes	123 (12.2)
	No	882 (87.8)
Other comorbidity	Yes	21 (2.1)
	No	984 (97.9)

**Figure 1:**
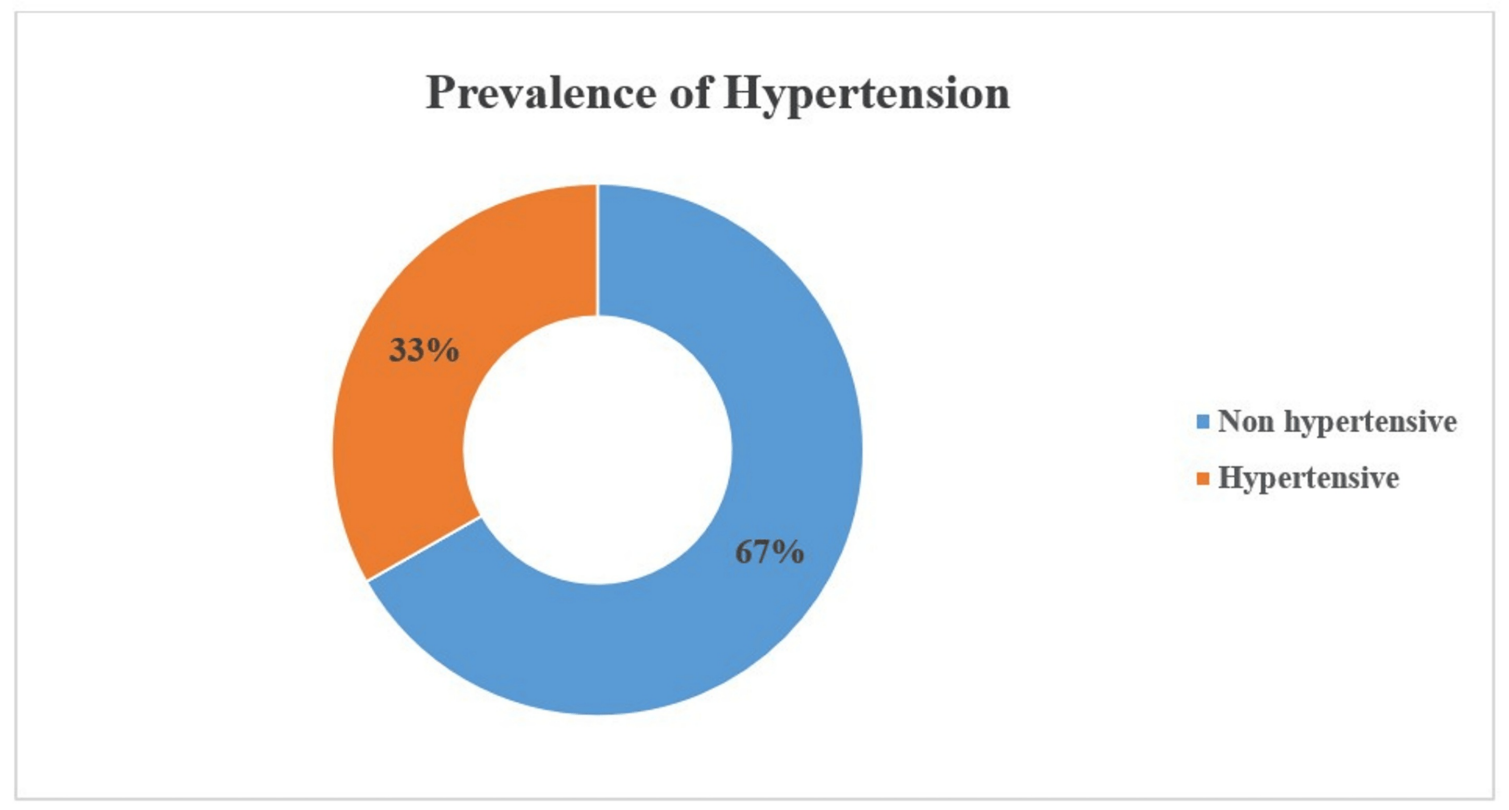
Prevalence of hypertension (study participants with raised blood pressure and/or on antihypertensive medication; n = 1,005)

**Figure 2:**
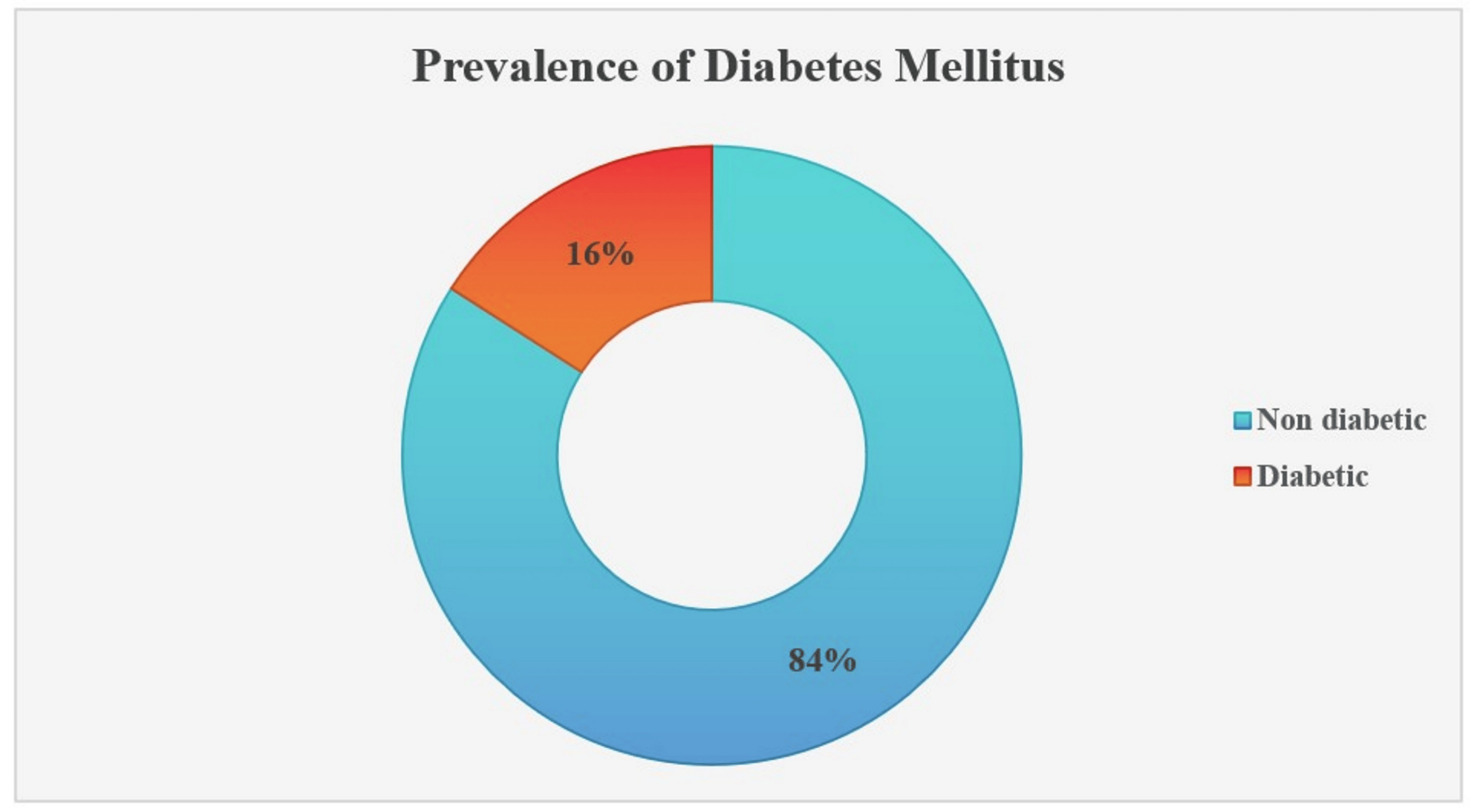
Prevalence of diabetes mellitus (study participants with elevated blood sugar and/or on anti-diabetic medication; n = 1,005)

The prevalence of hypertension and diabetes was 33% (95% CI: 30-36%) and 16% (95% CI: 14-18%), respectively (Figure 1 and Figure 2). The median systolic blood pressure was 126 mmHg (IQR: 110-140 mm Hg), and the median diastolic blood pressure was 80 mmHg (IQR: 70-90 mmHg). The study participants' median random blood sugar level was 121 mg/dL (IQR: 102.0-151.5 mg/dL).

Among the study participants who were found to be hypertensive in the study, more than 33.8% of the study participants were not on any antihypertensive medications. Among those who were on antihypertensive medications, 34.4% did not have their blood pressure under control (≤140/90 mmHg).

Age differences between hypertensive and non-hypertensive were statistically significant (Mann-Whitney U = 57,044, z = -12.71, p < 0.001). We also found statistically significant age differences between individuals with diabetes and those without diabetes (Mann-Whitney U = 43,515, z = -7.16, p < 0.001).

Among the study participants who were found to be diabetic, 23.1% were not on any anti-diabetic medications. Among the study participants on anti-diabetic medications, almost half (48.8%) did not have their blood sugar levels under control.

Age, alcohol consumption, and diabetes are the significant predictors of hypertension, whereas age and hypertension are the significant predictors of diabetes on multivariable logistic regression (p-value < 0.05, Table 2 and Table 3).

**Table 2:** Association between hypertension and key demographic and lifestyle factors (n = 1,005) *Shows statistical significance at p < 0.05 CI, confidence interval; Ref, reference

Independent variables		Hypertension		Crude odds ratio (95% CI)	p-value	Adjusted odds ratio (95% CI)	p-value
		No (%)	Yes (%)				
Age (years)	-	-	-	1.07 (1.06-1.08)	<0.001*	1.06 (1.05-1.08)	<0.001*
Gender	Male	216 (64.3)	120 (35.7)	1.18 (0.89-1.55)	0.23	1.16 (0.77-1.75)	0.469
	Female	455 (68)	214 (32)	Ref		Ref	
Alcohol consumption	Yes	11 (50)	11 (50)	2.04 (0.87- 4.76)	0.09	2.98 (1.13-7.85)	0.026*
	No	660 (67.1)	323 (32.9)	Ref		Ref	
Tobacco use	Yes	154 (57.7)	113 (42.3)	1.71 (1.28-2.29)	<0.001*	1.21 (0.87-1.69)	0.255
	No	517 (70.1)	221 (29.9)	Ref		Ref	
Family history of hypertension/diabetes	Yes	28 (73.7)	10 (26.3)	0.70 (0.34- 1.47)	0.35	1.14 (0.50-2.59)	0.746
	No	643 (66.5)	324 (33.5)	Ref		Ref	
Employed	No	393 (64.6)	215 (35.4)	1.27 (0.97-1.67)	0.07	1.12 (0.75-1.68)	0.571
	Yes	278 (70.0)	119 (30.0)	Ref		Ref	
Diabetes mellitus	Yes	67 (41.9)	93 (58.1)	3.47 (2.45 - 4.92)	<0.001*	2.55 (1.75-3.70)	<0.001*
	No	604 (71.5)	241 (28.5)	Ref		Ref	

**Table 3:** Association between diabetes mellitus and key demographic and lifestyle factors (n = 1,005) *Shows statistical significance at p < 0.05 CI: confidence interval, Ref: reference

Independent variables		Diabetes mellitus		Crude odds ratio (95% CI)	p-value	Adjusted odds ratio (95% CI)	p-value
		No (%)	Yes (%)				
Age (years)	-	-	-	1.04 (1.03-1.06)	<0.001*	1.03 (1.01-1.04)	<0.001*
Gender	Male	291 (86.6)	45 (13.4)	0.74 (0.51-1.08)	0.12	0.81 (0.48-1.34)	0.417
	Female	554 (82.8)	115 (17.2)	Ref		Ref	
Alcohol consumption	Yes	20 (90.9)	2 (9.1)	0.52 (0.12-2.25)	0.38	0.67 (0.14-3.14)	0.621
	No	825 (83.9)	158 (16.1)	Ref		Ref	
Tobacco use	Yes	211 (79.0)	56 (21.0)	1.61 (1.12-2.32)	0.009*	1.37 (0.92-2.04)	0.118
	No	634 (85.9)	104 (14.1)	Ref		Ref	
Family history of hypertension/diabetes	Yes	32 (84.2)	6 (15.8)	0.99 (0.40-2.40)	0.98	1.35 (0.52-3.45)	0.532
	No	813 (84.1)	154 (15.9)	Ref		Ref	
Employed	No	493 (81.1)	115 (18.9)	1.82 (1.25-2.64)	0.001*	1.41 (.85-2.32)	0.179
	Yes	352 (88.7)	45 (11.3)	Ref		Ref	
Hypertension	Yes	241 (72.2)	93 (27.8)	3.47 (2.45-4.92)	<0.001*	2.57 (1.77-3.75)	<0.001*
	No	604 (90)	67 (10.0)	Ref		Ref	

## Discussion

This study focuses on an urban slum, highlighting the health challenges faced by underprivileged communities. The study is based on a population-based screening register that comprises individuals screened for hypertension and diabetes over one year, and it has a large sample size. Missing or incomplete data were excluded from the analysis. The study offers vital insights into the epidemiology of hypertension and diabetes mellitus, which is crucial for developing effective public health policies to mitigate the burden of NCDs in vulnerable populations.

We found the prevalence of hypertension, which includes participants with raised blood pressure and/or on antihypertensive medications, was 33%, which is higher than other studies, implying that urban slum communities may encounter more health issues due to socioeconomic and cultural conditions. A systematic review conducted in 2014 reported a pooled prevalence of hypertension of 29.8% [11]. A similar result was reported by the NFHS-5, where the prevalence of hypertension was 28.1% [12]. A study by Murarkar et al. in 2023 found the prevalence of hypertension to be 15.36% [13]. Similarly, Kanungo et al. found a prevalence of 26.04% [14].

In our study, among hypertensives, 33.8% were not on any antihypertensive medications. A study by Kanungo et al. found that 83.86% of cases of hypertension go untreated among adults in Malda district, West Bengal [14]. A systematic review by Anchala et al. in 2014 found that only 42% of Indians know their hypertensive status [11]. According to NFHS-5 findings, only 36.9% of hypertensives were diagnosed with hypertension [12]. The difference in our study could be attributed to variations in healthcare availability, health awareness in the study area, and differences in operational definitions. According to a study by Agrawal et al., the prevalence of undetected hypertension among older persons in India is 21.3% [15]. A study by Appadurai et al. in 2023 found the prevalence of undiagnosed hypertension to be 36.9% [16]. A study by Shukla et al. found the prevalence of undiagnosed hypertension to be 26% [17].

We also found that of those who were on antihypertensive medications, more than one-third did not have their blood pressure under control. Anchala et al. found that 20.2% of hypertensive patients on medications in urban India do not have their blood pressure under control [11]. As per NFHS-5, 47.5% of individuals on antihypertensive medications do not have their blood pressure under control [12]. A study by Hirani et al. in 2024 in Gujarat found the prevalence of uncontrolled hypertension to be 60.2% [18]. The difference in our study could be due to variations in setting, healthcare availability, and awareness.

Our findings showed significant associations of hypertension with age, alcohol consumption, and diabetes mellitus. Older age significantly increases the risk of hypertension due to several interconnected physiological and lifestyle changes. As age increases, blood vessels become stiffer and less elastic, resulting in increased vascular resistance and blood pressure. Hormonal changes disrupt blood pressure regulation, leading to sodium retention and vasoconstriction. Additionally, the sensitivity of baroreceptors declines with age, thereby impairing the body's ability to maintain stable blood pressure. Physical inactivity and dietary shifts further worsen these effects, making older adults more susceptible to hypertension [19]. Similar results are found in other studies [11,13-15].

Alcohol consumption also drastically raises the risk of hypertension through several mechanisms. Excessive alcohol intake can lead to vasoconstriction, causing blood vessels to narrow and increasing vascular resistance, which raises blood pressure. Additionally, alcohol disrupts the balance of electrolytes and hormones that regulate blood pressure, resulting in sodium retention and fluid overload. Moreover, heavy drinking often correlates with other unhealthy lifestyle choices, such as poor diet and reduced physical activity, further worsening hypertension risk [20]. Other studies reported similar results [11,13,15].

Diabetes and hypertension are closely linked, with each condition serving as a significant risk factor for the other. Both conditions are part of metabolic syndrome, involving insulin resistance and inflammation that can damage blood vessels. Elevated blood glucose levels lead to endothelial dysfunction, which impairs blood vessel relaxation and increases vascular resistance. Additionally, diabetes often causes changes in kidney function that result in increased sodium retention, raising blood pressure. Furthermore, diabetes is commonly associated with obesity and a sedentary lifestyle, which further heightens the likelihood of hypertension [2,21,22].

We found a 16% diabetes prevalence in this study. The NFHS-5 data also show 14.6% of diabetes prevalence in urban areas, supporting our study [12]. Reasons for higher prevalence in urban areas may be lifestyle changes, dietary habits, and environmental influences. Urban areas often experience increased consumption of processed and high-calorie foods, leading to obesity, which is a significant risk factor for diabetes. Additionally, sedentary lifestyles are more prevalent in urban settings, often due to longer commutes and a reduction in physical activity. Residing in urban areas is also associated with higher stress levels, which can adversely affect metabolic health. Furthermore, limited access to healthcare services for lower-income urban residents can result in delayed diagnosis and management of diabetes. These combined factors contribute to the rising rates of diabetes observed in urban populations compared to their rural counterparts [23,24].

Among study participants diagnosed with elevated blood sugar levels, more than one-fifth (23.1%) were not on anti-diabetic medications. A study by Claypool et al. found that at least 42% of Indians remain unaware of their diabetic status [25]. Among the study participants who were on anti-diabetic medications, almost half (48.8%) of them did not have their blood sugar levels under control. A study by Anusuya et al. found that 65.4% of known cases of diabetes mellitus had their blood sugar level not under control [26]. A study by Ismail et al. in 2024 found the burden of uncontrolled diabetes in 75.6% of patients. The reasons for such high uncontrolled diabetes were lack of knowledge regarding blood sugar testing, irregular testing, and lack of physical activity [27]. Age was significantly associated with diabetes. Other studies reported similar results [8,28].

Strengths and limitations

The study highlights gaps in healthcare interventions that can be further explored in future research. The study was conducted in an urban slum with adequate sample size. However, this study has a few limitations. The study is record-based, and selection bias may affect the results. The accuracy of the findings also depends on how well the secondary data was collected. There may be limited generalizability of findings beyond the study area. Being a retrospective study, the causality of risk factors cannot be inferred. Due to limitations imposed by the records, some potential confounding variables cannot be explored. Random blood sugar levels were used in the survey, which have lower sensitivity and specificity compared to HbA1C. This may affect study results. Random blood sugar levels may be considered in further studies to validate the results for use in low-resource settings, eliminating the need for fasting conditions.

## Conclusions

The study highlights the vulnerability of populations living in urban slums to noncommunicable diseases such as hypertension and diabetes mellitus. Many individuals with hypertension or diabetes mellitus are unaware of their disease status, and even those who are aware may not have their clinical parameters under control. The study identified key risk factors of advancing age, alcohol consumption, and diabetes mellitus for hypertension, while age and hypertension were significant predictors of diabetes mellitus. Targeted interventions are needed to control hypertension and diabetes mellitus. Health programs should focus on increasing awareness about lifestyle factors, enhanced screening, and treatment adherence to control the modern pandemic of hypertension and diabetes mellitus.
